# Correction of Vitamin D Deficiency Improves PTSD Symptoms in Gulf War Veterans

**DOI:** 10.3390/brainsci15111135

**Published:** 2025-10-23

**Authors:** Chandrasekhar Kesavan, Donna D. Strong, Richard M. Strong

**Affiliations:** 1 Department of Gastroenterology, VA Loma Linda Healthcare System, Loma Linda, CA 92357, USA; 2 Department of Medicine, Loma Linda University, Loma Linda, CA 92354, USA; 3 Musculoskeletal Disease Center, VA Loma Linda Healthcare System, Loma Linda, CA 92357, USA; donna.strong2@va.gov

**Keywords:** post-traumatic stress disorder (PTSD), traumatic brain injury (TBI), vitamin D deficiency (VDD), Gulf War Veterans (GWV)

## Abstract

Gulf War Veterans (GWVs) presenting with irritable bowel syndrome-diarrhea (IBS-D) often exhibit concurrent post-traumatic stress disorder (PTSD) or traumatic brain injury (TBI). All Veterans’ Affair physicians are required to assess symptoms of depression, anxiety, and suicide ideation by maintaining yearly training. In a previous study for IBS-D (3), we identified significant vitamin D deficiency (VDD), with an average level of 19 ng/mL. This includes those with and without PTSD, TBI, showing depression and anxiety symptoms. Since VDD is associated with PTSD, and all veterans not on supplementation were found to be VDD (>90%) at our facility, we investigated a possible association between VDD and these neuropsychological conditions. While age and body mass index (BMI), seasons, and demographic locations are known to affect vitamin D levels, we found no correlation between these factors and VDD in the PTSD group and those with IBS-D. In the TBI group, VDD did correlate with BMI. Multiple deployments appeared to have a minor negative effect on vitamin D levels (a 11–13% contribution) in veterans with either PTSD or TBI. Although these veterans showed signs of inflammation with elevated minor C-reactive protein (CRP) levels (4.7 mg/L), there was a negative correlation between CRP and vitamin D to suggest that inflammation is not the primary cause of PTSD or TBI. Following daily vitamin D treatment, vitamin D levels returned to a normal average of 30 ng/mL (less than 30 ng/mL is abnormal). Treatment had no effect on serum calcium levels, but did lead to a resolution of depression, anxiety, TBI, and PTSD symptoms in the majority of patients. These findings suggest that correcting VDD in GWVs visiting GI clinics with co-occurring PTSD and TBI had reduced psychological symptoms. Replacing vitamin D is a simple strategy to implement, rather than increasing neurotrophic medications in some patients.

## 1. Introduction

Gastrointestinal (GI) problems are a well-documented health issue for military personnel following deployment [1,2,3,4]. While historic conflicts are associated with peptic ulcers and infections, the nature of modern warfare has shifted the disease spectrum [5]. Today, non-battle injuries, particularly non-battle neuropsychological disorders (NBNPDs) and digestive diseases, are predominant [6,7,8]. Following the first Gulf War, veterans began reporting a constellation of symptoms, later termed Gulf War Syndrome, in which chronic inflammatory bowel syndrome diarrhea (IBS-D) was a key clinical feature [9,10].

Many of these same veterans also present to both mental health and GI clinics. The mental health clinics deal with primarily managing mental health issues with counseling and often with prescriptions, but the GI clinics address both GI complaints of abdominal pain, diarrhea, and fatigue, as well as the stability of neuropsychological conditions such as post-traumatic stress disorder (PTSD) and non-injury traumatic brain injury (TBI). The GI clinics primarily address the non-psychological complaints but do assess the psychological complaints as all Veterans’ Affair (VA) physicians are expected to do with yearly retraining in order to help decrease suicide incidence. The relationship between these neuropsychological disorders and chronic diarrhea, however, remains unclear. Our previous research indicated that vitamin D deficiency (VDD), rather than infection or inflammation, was a possible cause for IBS-D in this group [11,12]. Other studies have since corroborated that VDD is common in patients with irritable bowel syndrome and that supplementation can improve symptoms not only of diarrhea but also on the functions of brain health [13,14,15].

The role of vitamin D extends beyond gut health, as it is also an important regulator of inflammation and other anabolic processes essential for homeostasis [16,17,18]. Separate lines of research have established a strong link between VDD and adverse neuropsychological outcomes. For example, a meta-analysis of over 300,000 veterans associated VDD with increased suicide risk [13], and other studies showed that VDD can exacerbate neuropsychological symptoms following an injury [14,15]. Based on these converging lines of evidence, as part of managing IBS-D, we undertook a retrospective study to clarify the role of vitamin D in male Gulf War Veterans (GWV) who presented with GI complaints together with a diagnosis of PTSD or TBI at the VA Loma Linda Healthcare System.

## 2. Materials and Methods

### 2.1. Patient Cohort

From a pool of 4221 patients seen in the Digestive Disease Clinic at the VA Loma Linda Healthcare System between (VALLHS) September 2014 and September 2020, we identified 55 GWVs with PTSD and 22 GWVs with TBI in the cohort studied for IBS-D [3]. All of the psychological diagnoses were made by mental health providers where they were diagnosed and managed, but patient mental health complaints were also assessed at each visit to the gastroenterology clinics, as VA physicians are required to assess symptoms of depression, anxiety, and suicide ideation by maintaining training yearly (training management system (TMS)). Based on this, one investigator (RMS) followed these patients prospectively and, consistently asking about their mental health symptoms, reviewed all prescriptions for mental health issues, but did not change prescriptions from mental health prescribers. We analyzed the data retrospectively; problem lists for all VA patients were updated to ICD-10 diagnoses.

### 2.2. Patient Evaluation

All patients underwent a comprehensive GI evaluation, including upper and lower endoscopies with biopsies, duodenal aspirates for culture, and stool analysis for calprotectin, parasites, and enteric pathogens. The evaluation also included radiographic series, computed tomography (CT) scans, and routine laboratory panels (tissue transglutaminase, immunoglobulins (IgA), C-reactive protein (CRP), erythrocyte sedimentation rate (ESR), complete blood count (CBC), thyroid, and chemistry). No cortisol or neuroinflammatory markers were obtained by either the gastroenterologist or the mental health clinic. A psychiatrist or psychologist in the mental health clinic concurrently followed the patients, but their notes were not reviewed, but the gastroenterologist seeing the patients re-assessed their mental health status. The mental health notes are restricted to the mental health providers and not available to other practitioners.

### 2.3. Data Collection

We collected data on patient age, gender, number of Gulf War deployments, PTSD/TBI diagnosis, and the number of bowel movements before and after vitamin D treatment (3000–5000 units daily), as well as whether PTSD or TBI improved (increased sense of wellbeing and energy levels, or reduced depression, anxiety, and brain fog). The diagnosis of VDD (deficient (<20 ng/mL), insufficient (20–30 ng/mL), and sufficient (>30 ng/mL)) [19] was made in each patient when they first visited the GI clinic.

### 2.4. Statistical Analysis

Data are presented as the mean ± standard deviation. We used Statistica 10 to perform Student’s *t*-tests and one-way paired *t*-tests. A *p*-value of less than 0.05 (*p* < 0.05) was considered statistically significant.

## 3. Results and Discussion

Many military personnel who served in the Gulf War experience a range of chronic health problems, most commonly chronic diarrhea (IBS-D) and neuropsychological disorders like PTSD and TBI. Our investigation into GWVs with this combination of symptoms revealed that their most salient laboratory finding was a significant vitamin D deficiency. The average vitamin D level in both the PTSD and TBI groups was 19 ng/mL, well below the 30 ng/mL threshold considered adequate for overall health homeostasis (Table 1). This deficiency was significant, regardless of a PTSD or TBI diagnosis, a finding consistent with reports in female veterans, which suggests VDD in this context is not dependent on gender [20].

Because vitamin D levels can be influenced by age and body mass index (BMI) [21,22,23], we performed a correlation analysis to assess their impact. For patients with PTSD, we found no significant correlation between vitamin D levels and either age or BMI (Figure 1). For patients with TBI, however, we observed a significant negative correlation (r = −0.40) between vitamin D and age, and a positive correlation (r = 0.20, *p* = 0.36) with BMI. The association with BMI in the TBI group may be due to the sedentary lifestyles common in these patients. Increased body fat can sequester this fat-soluble vitamin in adipose tissue, thereby reducing its bioavailability in the bloodstream.

We next assessed if deployment history affected vitamin D status, as multiple deployments could compound health issues. We found a positive correlation between vitamin D levels and the number of deployments, but the overall effect was minor, accounting for approximately 13% of the variance in vitamin D levels for GWVs with PTSD or TBI (Figure 2A). We were unable to determine what factors influenced this 13% variance in vitamin D levels because VDD is a worldwide problem with no clear explanation, despite variation in sunlight exposure, diet, and deployment. In 2014, only 40% of veterans were VDD; now, it is nearly 100% with supplementation and without regard to time spent outside.

Since both PTSD and TBI can trigger inflammatory responses [24,25,26,27,28], which could in turn lower vitamin D, we measured CRP, a standard inflammatory marker [29,30,31,32] which correlates well with the old traditional ESR. The veterans’ initial CRP levels were significantly elevated (mean 4.7 mg/L, *p* < 0.05) compared to the laboratory standard (<3.0 mg/L) (Figure 2B). However, a subsequent correlation analysis revealed a negative association between CRP and initial vitamin D levels (Figure 2C). This result suggests that while minor inflammation is present in these veterans, it is not the primary cause of their vitamin D deficiency.

Given the pronounced deficiency, 3000–5000 international units (iu) of vitamin D were prescribed daily, based on weight (weight > 150 lbs usually on 5000 iu for capsules in oil, not pills, taken in the morning). Since vitamin D is a fat-soluble vitamin, pills must be taken with a fatty meal, while capsules in oil can be taken by themselves. After three months of supplementation, follow-up testing showed that vitamin D levels in both the PTSD and TBI groups were restored to a normal average of 30 ng/mL. There were multiple assessments for vitamin D levels, negating effect of the time of year. This treatment dosage did not cause hypercalcemia [33], a potential side effect of vitamin D supplementation. We found the serum calcium levels were within the acceptable normal range and there was in GWV with GI compliant with PTSD or TBI (Figure 2D). Furthermore, negative correlations suggest that the vitamin D dosage used in this study did not significantly alter the calcium levels (Figure 3). Following the restoration of their vitamin D levels, a remarkable clinical improvement was observed. Based on physician observation and patient questioning during follow-up visits, approximately 80% of the GWVs with PTSD reported being less depressed and feeling healthier overall with less anxiety (Figure 2E). Interestingly, post-treatment vitamin D levels showed a new positive correlation with age (r = 0.15, PTSD group), suggesting that the prescribed dose was effective at restoring homeostasis across different age groups. However, to validate this pilot data, future studies will include a larger sample size to achieve increased significance.

In the PTSD cohort with vitamin D deficiency (VDD), 58 patients were evaluated. Of these, 16 patients received a combination of vitamin D supplementation along with anti-depressant, anti-anxiety, or both types of psychotropic medications. Following treatment, 11 of these 16 patients showed an increase in serum vitamin D levels to the range of 25–50 ng/mL, while 5 patients remained vitamin D deficient (10–18 ng/mL). This observation suggests that the combined treatment may have contributed to symptom improvement in a subset of patients. However, physician-based assessments indicated that the most notable improvement in PTSD symptoms was observed in patients who received only vitamin D supplementation, without changing anti-depressant or anti-anxiety medications. This suggests that vitamin D alone may play a primary role in alleviating PTSD symptoms in some individuals. The lack of vitamin D response in the subgroup receiving psychotropic medications could be attributed to two potential mechanisms: (1) genetic variations, such as mutations in the vitamin D response element (VDRE) in the promoter region, potentially impairing vitamin D signaling; or (2) pharmacological interactions wherein psychotropic medications may indirectly alter vitamin D metabolism or absorption. The fact that correcting the VDD helps PTSD, TBI, depression, and anxiety is amazing and represents a simple treatment without adding another neuropsychiatric drug.

## 4. Conclusions

In summary, this study provides strong evidence for the role of vitamin D in the health of Gulf War Veterans suffering from comorbid GI and neuropsychological disorders. It clearly supports the association of mental health with vitamin D levels. We found that (1) GWVs with PTSD and TBI have a significant vitamin D deficiency. (2) This deficiency is not primarily driven by age, BMI, or underlying inflammation. (3) While multiple deployments contribute slightly to the deficiency, they are not the main factor. (4) Restoring vitamin D levels to normal with a daily dose of 3000–5000 units is safe and does not adversely affect calcium levels. (5) Most importantly, correcting vitamin D deficiency led to resolution of depression and anxiety symptoms observed in patients with excessive bowel movements.

The study has limitations: (1) the relatively small sample size used for correlation analyses. Future prospective studies using a placebo and involving larger, more diverse populations could provide greater insight into which veterans might benefit most from vitamin D screening and supplementation. Nonetheless, our findings strongly suggest that assessing and correcting vitamin D status should be considered a component of care for this patient population. (2) Fewer veterans might be prescribed less anti-depressants and anti-anxiety medications. We predict that some of the reduction in PTSD symptoms is likely due to the effects of these anti-depressants but enhanced with supplementing vitamin D.

### Future Directions

Veterans with psychological symptoms from PTSD or mild TBI in combination with and without non-infectious diarrhea need to be assessed for VDD (<20 ng/mL) and CRP (>3.8 mg/L). If these lab values are below normal, they need to be placed under vitamin D supplementation (3000 units/day for 3 months) in combination with vitamin K for faster absorption to reduce the symptoms of PTSD related to diarrhea or other factors, for example, number of deployments. Future studies with larger populations are necessary to differentiate between the effects of vitamin D and the effects of antipsychotic agents on reducing the PTSD symptoms. (3) For the TBI group, we were able to find 22 patients that met our inclusion criteria in our local CPRS electronic database. Since all these patients showed improved vitamin D levels after supplementation and improved psychological symptoms, we believe these pilots findings are important for future mechanistic interpretations. (4) Not being able to review the notes in the mental health clinics might have overestimated the benefits that were observed in the GI clinics, but VA physicians are trained yearly to also make these assessments and, other than supplementation of vitamin D, no other changes were made in the neurotropic medications. Future studies will include a mental health provider to compare clinical notes with a scale to assess TBI, PTSD, depression, and anxiety. Monitoring pharmacological drug levels before and after vitamin D supplementation might shed light on clinical effects as supplementation might increase drug levels to improve mental health by displacing protein-bound medications. And clarify how vitamin D supplementation might be working.

## Figures and Tables

**Figure 1:**
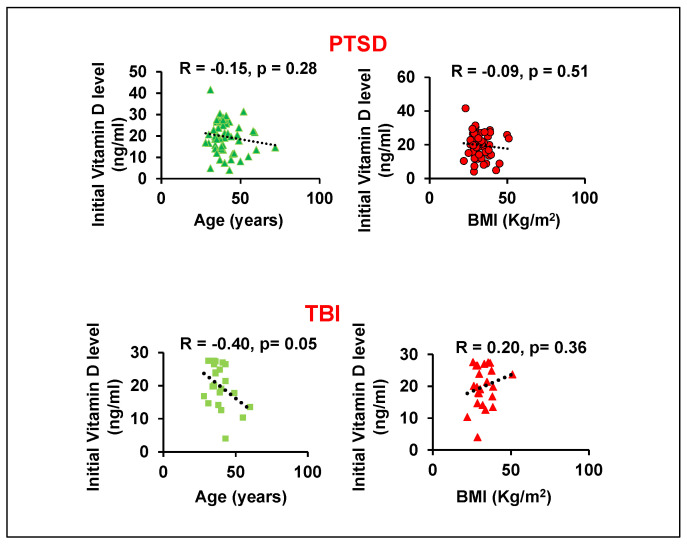
Correlation analysis between initial vitamin D levels with age or BMI in GWVs with PTSD and TBI.

**Figure 2:**
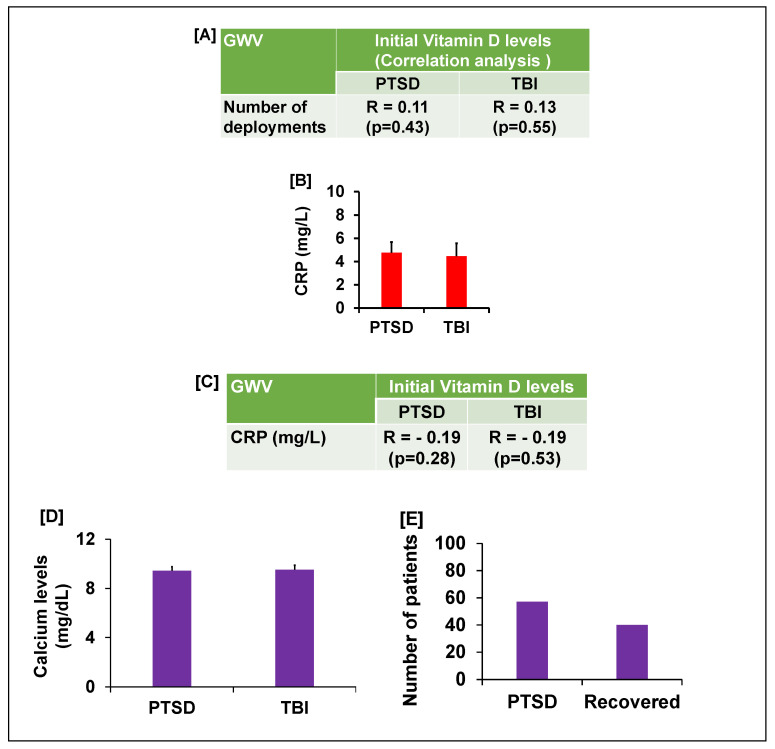
(**A**) The table shows the correlation analysis between the number of deployments and initial vitamin D levels in GWVs with PTSD or TBI. (**B**) CRP levels and (**C**) table shows the correlation between initial vitamin D levels with CRP (inflammatory marker) in GWVs with PTSD and TBI. (**D**) Vitamin D supplementation did not cause hypercalcemia in GWV with GI complaints and PTSD/TBI. (**E**) 80% GWV with GI complaints and PTSD reported reduced psychological symptoms.

**Figure 3:**
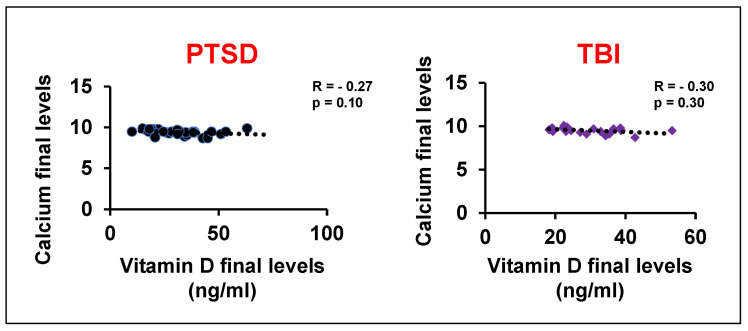
Correlation analysis of vitamin D levels with blood calcium levels after treatment in GWVs with PTSD and TBI.

**Table 1:** One paired *t*-test of vitamin D levels compared to the standard vitamin D range in the GWVs with PTSD and TBI.

	Vitamin D (ng/mL)							
	Mean	Std.Dv.	N	Std.Err.	Reference Constant	t-Value	df	*p*
PTSD	19.65	7.43	55	1.00	25	−5.32	54	0
TBI	19.89	6.35	23	1.32	25.0	−3.85	22	0

## Data Availability

The data presented in this study are available on request from the corresponding author due to ethical and institutional restrictions.
